# Supplementary material for: A Novel Unorthodox Dimeric Primary Enoyl-CoA Reductase Structure
Source: bioRxiv. 2025 Oct 17:2025.10.17.683039. Preprint. [Version 1] doi: 10.1101/2025.10.17.683039 (PMC12632816; doi:10.1101/2025.10.17.683039)
Supplement: Supplement 6 [file media-6.pdf]

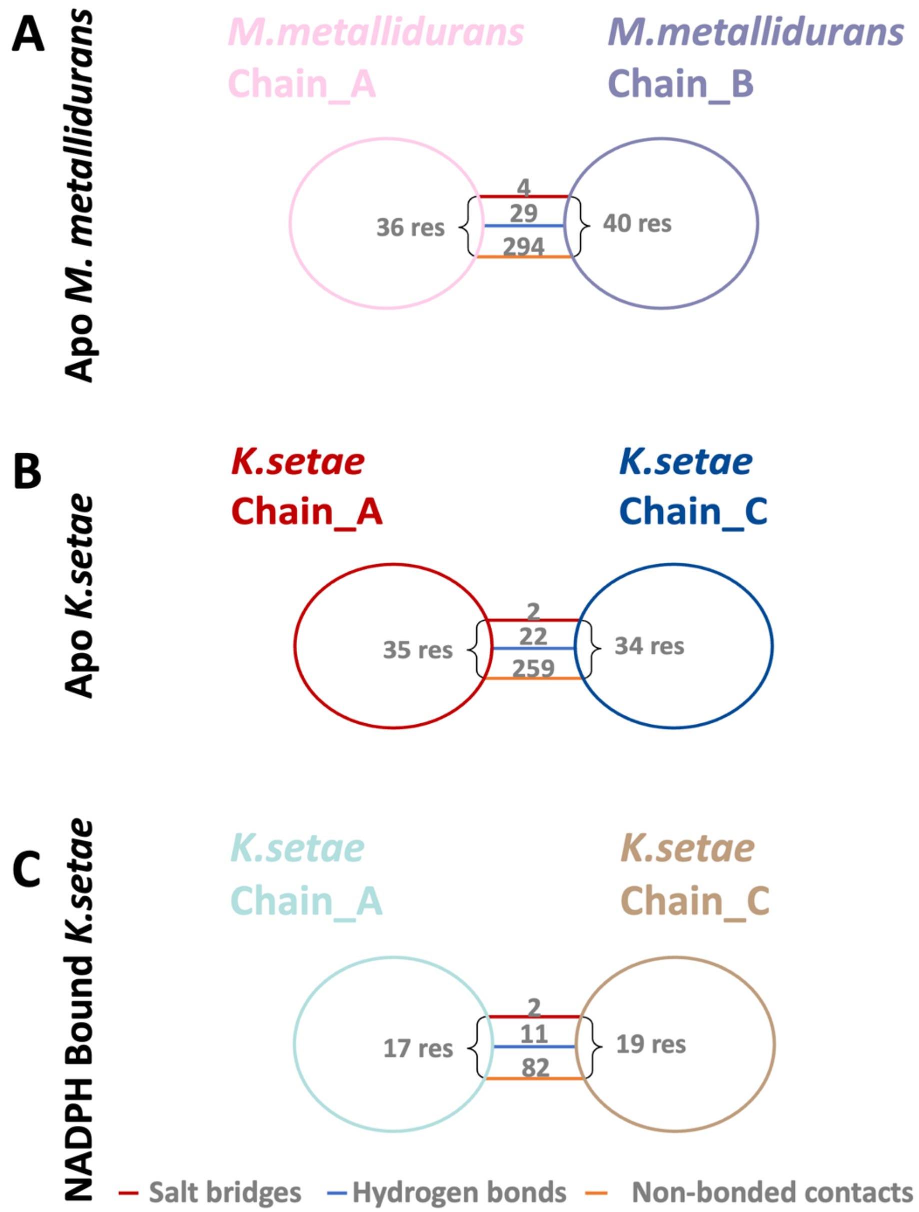

Supplementary Figure 6: Structural Analysis of Dimer and Tetramer Interfaces, **a)** Shows the numbers of salt bridges, hydrogen bonds, and non-bonded contacts between homologs in the Apo *M. metallidurans* dimer structure. You can see these interactions in the interaction residues given in Scheme 1. **b)** Shows the number of salt bridges, hydrogen bonds, and non-bonded contacts in the dimer portion of the Apo *K. Setae* tetramer structure (PDB ID: 6NA3). You can see these interactions in the interaction residues given in Scheme 2. **c)** Shows the number of salt bridges, hydrogen bonds, and non-bonded contacts in the dimer portion of the NADPH Bound *K. setae* tetramer structure (PDB ID: 6NA6). You can see these interactions in the interaction residues given in Scheme 3.

Note: Each panel details the interactions between two chains (chain_A and chain_B or chain_C) and categorizes them into salt bridges (red), hydrogen bonds (blue), and non-bonded contacts (orange). Additionally, the number of residues involved in the contact surface for each chain is indicated.
